# Defense Profiles in Adaptation Process to Sport Competition and Their Relationships with Coping, Stress and Control

**DOI:** 10.3389/fpsyg.2017.02222

**Published:** 2017-12-19

**Authors:** Michel Nicolas, Guillaume Martinent, Martin Drapeau, Khadija Chahraoui, Philippe Vacher, Yves de Roten

**Affiliations:** ^1^Laboratory Psy-DREPI (EA 7458), University of Bourgogne Franche-Comté, Dijon, France; ^2^Laboratory of Vulnerabilities and Innovation in Sport (EA 7428), University of Claude Bernard Lyon 1 – University of Lyon, Villeurbanne, France; ^3^Psychotherapy Process Research Group, Department of Educational and Counselling Psychology, McGill University, Montréal, QC, Canada; ^4^University Institute of Psychotherapy, University Hospital Center, University of Lausanne, Lausanne, Switzerland

**Keywords:** defense mechanisms, stress, clusters analysis, sport competition, adaptation

## Abstract

The purpose of this study was to identify the potentially distinct defense profiles of athletes in order to provide insight into the complex associations that can exist between defenses and other important variables tied to performance in sports (e.g., coping, perceived stress and control) and to further our understanding of the complexity of the adaptation process in sports. Two hundred and ninety-six (*N* = 296) athletes participated in a naturalistic study that involved a highly stressful situation: a sports competition. Participants were assessed before and after the competition. Hierarchical cluster analysis and a series of MANOVAs with *post hoc* comparisons indicated two stable defense profiles (high and low defense profiles) of athletes both before and during sport competition. These profiles differed with regards to coping, stress and control. Athletes with high defense profiles reported higher levels of coping strategies, perceived stress and control than athletes with low defense profiles. This study confirmed that defenses are involved in the psychological adaptation process and that research and intervention should not be based only on coping, but rather must include defense mechanisms in order to improve our understanding of psychological adaptation in competitive sports.

## Introduction

Before, during and after each competition, athletes cope with specific physical, technical, and psychological demands (Cerin et al., 2000). With the exception of some more recent studies (Cramer, 1991; Endler and Parker, 2002; Hoar et al., 2006), researchers have generally failed or neglected to examine how individuals respond to stressful or challenging situations with regards to the combination of coping strategies, defense mechanisms, and other adaptation processes that they might use. Past studies have focused predominantly on coping and have neglected defense mechanisms (Hoar et al., 2006), despite evidence indicating that they are important to understanding the adaptation process (Fulde et al., 1995; Erickson et al., 1997; Bouchard and Thériault, 2003; Nicolas and Jebrane, 2008, 2009; Nicolas et al., 2013, 2015). Adaptation is a polysemic and interdisciplinary concept which could be considered as a dynamic process of constant adjustment to environments encompassing all changes in physical, social and psychological demands and constraints (Cramer, 1991, 2000, Lazarus, 1991, 2000; Levine and Ursin, 1991; Cerin et al., 2000; Lucas et al., 2003; Ursin and Eriksen, 2004; Tamminen and Crocker, 2014).

Coping originally stemmed from the construct of defense mechanisms when in the late 1960’s, researchers considered adaptive defense mechanisms as a coping strategy (Cramer, 1998). Based on Lazarus and Folkman (1984) seminal model of psychological stress, coping is defined as the “constantly changing cognitive and behavioral efforts to manage specific external and/or internal demands that are appraised as taxing or exceeding the resources of the person.” In this theoretical framework, coping strategies are related to the appraisal of a situation by an individual and to his or her determination as to whether or not they have the resources to adapt to the situation (Folkman, 1984). Among the several classifications of coping strategies proposed (Skinner et al., 2003), coping strategies have been classified into a three second-order meaningful sets of coping dimensions addressed by the approach of hierarchical models of coping (Gaudreau and Blondin, 2002; Skinner et al., 2003). Task-oriented coping (TOC) represents strategies facing directly to a stressful situation and the resulting thoughts and affects, as opposed to Disengagement-oriented coping (DgOC) that refers to withdrawal and to not actively striving toward the achievement of desirable outcomes. In recent years, a third dimension, distraction-oriented coping (DtOC), emerged to apprehend the strategies used to focus on external and internal stimuli unrelated to the stressful situation.

Defense mechanisms are a core concept in the psychodynamic literature (Cramer, 1998), and one of the rare psychodynamic constructs to lend itself to empirical study (Despland et al., 2001). In the Diagnostic and Statistical Manual of Mental Disorders (DSM III-R; American Psychiatric Association, 1987), defense mechanisms are defined as automatic psychological processes that protect the individual against anxiety and from the awareness of internal or external dangers or stressors. In the last two decades, this construct has also been related to, and compared with, other similar constructs such as adaptation and coping (Bonsack et al., 1998; Cramer, 2000; Bouchard and Thériault, 2003). Even if the difference between coping and defenses remains sometimes unclear in the literature, it is generally believed that coping reflects competence-related functioning whereas defenses are related to internal determinants of functioning (Kramer, 2010). Defenses are considered to be more unconscious and predominantly directed toward inner conflicts, whereas coping is presumed to be relatively conscious and oriented toward outer stressors and adaptation to reality (Parker and Endler, 1996; Cramer, 1998). However, studies show that even though coping and defenses have common and unique features, these two concepts are two aspects of the same process: adaptation (Fulde et al., 1995; Erickson et al., 1997; Bouchard and Thériault, 2003; Nicolas and Jebrane, 2008; Nicolas et al., 2015).

The different defense mechanisms can be grouped into a meaningful and parsimonious set of dimensions or levels based on their level of maturity (Vaillant, 1977, 1994; Thygesen et al., 2008). Based on Vaillant’s model (Vaillant, 1977), defenses are grouped as mature defenses (e.g., using problem-solving), intermediate defenses (e.g., expressing altruistic and prosocial behaviors or attitudes), and immature defenses (e.g., expressing affect through withdrawal or acting-out). Research on individual defense mechanisms has shown that, for example, the mature defenses, namely, anticipation and humor are related to desirable outcomes such as performance, whereas the immature defenses, namely, dissociation and projection are related to negative consequences such as ill-being, depression or non-adaptation (Bouchard and Thériault, 2003; Nicolas et al., 2013, 2015).

While such research efforts are indeed important, most previous studies have unfortunately focused on the bivariate relationships that exist between defense mechanisms and other variables and have neglected the nature of defenses when individuals endorse multiple defense mechanisms simultaneously. Indeed, different defenses can coexist to a varying degree within each individual in a given situation. For this reason, the primary goal of this study was to address this limitation and to examine a framework in which the use of several defenses is considered to generate multivariate defense profiles which may differ across individuals. In order to shed light on change in defense profiles over time, we also explored whether defense profiles fluctuate before (an anticipatory stage) and during (a performance stage) sport competitions. Sport competitions are achievement-related situations that place many demands on individuals (e.g., individuals must master technically demanding skills and demonstrate their competence relative to others) (Martinent et al., 2013). Such situations typically involve an anticipatory stage (i.e., preparation for action with mental, physical, technical, and tactical preparation) and a performance stage (i.e., task execution), each distinguished by a precise phenomenological experience with particular psychological, physical, and technical demands. Because these two moments place different demands on the individual, it is possible that the defense profiles of the athletes maybe different (Martinent and Nicolas, 2016).

Lazarus and Folkman’s psychological stress framework suggests that the interaction between the person and the environment is mediated by perceived stress referring to the stakes a person judged whether an encounter is irrelevant or stressful, and perceived control pertaining to the person’s judgment or belief to having individual resources to achieve the expected outcomes (Folkman, 1984). Nevertheless, the relationships between perceived stress and control on one hand, and defense mechanisms on the other hand have been neglected in sport settings in spite of their central role in adaptation process. Thus, exploring the relationships between athletes’ coping strategies and perceived stress and control and the within-person configurations of the different types of defense offer a strong heuristic to investigate defenses using a more holistic approach. Despite advances in understanding how specific defense profiles in sports may be associated with different coping responses, and perceived stress and control is of prime importance both theoretically and practically, this research area has received only sparse attention. Theoretically, this issue could help to better demarcate resemblances and divergences between coping and defenses in order to develop our insight of the mechanisms involved in adaptation process. Practically, providing some insights into the defense and coping relationships and their contribution in adaptation process might help practitioners and psychologists to adapt interventions to the needs of athletes characterized by particular defense profiles.

Cluster analysis aids in determining profiles with a person-centered approach which classifies participants using multivariate methods to gather subjects based on their characteristics (i.e., defense scores) by optimizing both the homogeneity of cases within a group and the heterogeneity between the clusters (Aldenderfer and Blashfield, 1984). In cluster analysis, another variable than the one used to generate the profile groups has upheld an effective method for endorsing this type of analysis (Aldenderfer and Blashfield, 1984; Martinent et al., 2013). To the best of our knowledge, no study to date has explored defense profiles that exist in achievement-related situations such as a sport competition. Another goal of this study was to examine whether subgroups of athletes (based on defense profiles) differ on coping, perceived stress and perceived control. We selected these variables in light of the central theoretical role of these constructs in the psychological adaptation process (Cramer, 2000; Lazarus, 2000; Kramer, 2010), and the empirical relationships that exist between defenses, coping, perceived stress and control in general psychology (Fulde et al., 1995; Erickson et al., 1997; Bouchard and Thériault, 2003), sport competition (Nicolas and Jebrane, 2008, 2009), or extreme situations (Nicolas et al., 2013, 2015). Further, we set out to test the following hypothesis: (a) athletes with high levels of mature defenses and low levels of immature defenses would indicate higher scores of TOC and perceived control and lower scores on stress; and that (b) athletes with low levels of task-oriented coping mature defenses and high levels of immature defenses would display lower scores of TOC and perceived control and higher scores on stress.

## Materials and Methods

### Participants

Two hundred and ninety-six French athletes (33% female; *M*_age_ = 21.61, *SD* = 6.32) participated in this study. Athletes were drawn from nine different sports: soccer (*n* = 23 females and 79 males), handball (*n* = 16 and 28), swimming (*n* = 19 and 18), basketball (*n* = 7 and 23), badminton (*n* = 6 and 22), cycling (*n* = 12 and 6), gymnastics (*n* = 12 and 4), athletics (*N* = 3 and 11), and tennis (*n* = 7 males). They participated in departmental (district, 9%), regional (provincial, 41%), national (42%) or international sport events (8%).

### Measures

The Defense Style Questionnaire is the most widely used self-report measure designed to assess conscious derivatives of defense mechanisms (Thygesen et al., 2008). In the current study, we used the French 60-item version exploring 30 defense mechanisms clustered in three higher-order factors as proposed by Thygesen et al. (2008) to be congruent with the Diagnostic and Statistical Manual of Mental Disorders (DSM III-R; American Psychiatric Association, 1987), classification of defense mechanisms. The three categories were mature (18 items), intermediate (22 items) and immature level (20 items) dimensions. Previous research has shown the validity and reliability of the DSQ-60 (Muris and Merckelbach, 1994; Bonsack et al., 1998; Thygesen et al., 2008; Petraglia et al., 2009; Drapeau et al., 2011). The scale is rated using a Likert type scale ranging from 1 (strongly disagree) to 9 (strongly agree). Cronbach alphas ranged from 0.71 to 0.81 (**Table 1**).

**Table 1:** Standardized and raw scores of defenses across the two clusters for the two waves.

	High defense profile				Low defense profile				α	*F* (1,294)	*p*	*η^2^*
	*Z* scores		Raw scores		*Z* scores		Raw scores					
	*M*	*SD*	*M*	*SD*	*M*	*SD*	*M*	*SD*				
T1	(*N* = 164)				(*N* = 132)							
Immature defenses	0.60	0.76	4.17	0.73	–0.75	0.71	2.87	0.68	0.77	243.89	<0.001	0.45
Intermediate defenses	0.62	0.67	4.58	0.63	–0.77	0.78	3.29	0.73	0.76	267.30	<0.001	0.48
Mature defenses	0.48	0.75	5.57	0.67	–0.60	0.95	4.61	0.85	0.71	118.07	<0.001	0.29

T2	(*N* = 183)				(*N* = 113)							
Immature defenses	0.55	0.76	4.04	0.75	–0.89	0.64	2.62	0.64	0.81	282.23	<0.001	0.49
Intermediate defenses	0.52	0.78	4.35	0.74	–0.83	0.71	3.08	0.67	0.79	222.43	<0.001	0.43
Mature defenses	0.49	0.71	5.42	0.68	–0.80	0.88	4.18	0.84	0.75	193.42	<0.001	0.40

The Perceived Stress Scale (Cohen and Williamson, 1988) (PSS) was used to measure perceived stress, and the Mastery Scale (Pearlin and Schooler, 1978) (MS) was used to measure perceived control. To shorten testing time, three items adapted from the PSS (“I felt nervous during the competition,” “I thought that the competition was a source of stress for me,” “I was worried because my performance during the competition could have negative consequences on me”) and three items from the MS (“I felt able to face to the stress competition,” “I got the resources to face to the constraints of the competition,” “I felt I was able to overcome the difficulties during the competition”) were used to measure the degree to which a sport’s competition is appraised as stressful and controllable. Each item was rated on a 7-point Likert scale ranging from 1 (strongly disagree) to 7 (strongly agree). Cronbach’s alphas ranged from 0.73 to 0.82 (**Table 2**).

**Table 2:** Comparison of external variables across the defense clusters for the two waves.

	High defense profile		Low defense profile		α	*F*(1,294)	*p*	*η^2^*
	*M*	*SD*	*M*	*SD*				
T1 Clusters	(*N* = 164)		(*N* = 132)					
T1 Task-oriented coping	2.72	0.62	2.37	0.60	0.88	24.91	<0.001	0.08
T1 Distraction-oriented coping	2.22	0.67	1.79	0.54	0.73	37.51	<0.001	0.11
T1 Disengagement-oriented coping	1.72	0.56	1.48	0.54	0.76	13.01	<0.001	0.04
T1 Perceived stress	3.45	1.36	2.79	1.13	0.73	20.10	<0.001	0.06
T1 Perceived control	5.18	1.15	4.86	1.29	0.82	5.10	0.02	0.02

T2 Clusters	(*N* = 183)		(*N* = 113)					
T2 Task-oriented coping	2.80	0.55	2.43	0.50	0.85	33.77	<0.001	0.10
T2 Distraction-oriented coping	1.97	0.71	1.44	0.45	0.79	50.24	<0.001	0.15
T2 Disengagement-oriented coping	2.19	0.79	1.78	0.65	0.80	22.59	<0.001	0.07
T2 Perceived stress	3.46	1.51	2.82	1.23	0.77	14.57	<0.001	0.05
T2 Perceived control	4.60	1.25	4.61	1.24	0.77	0.01	0.93	0.00

The Coping Inventory for Competitive Sport (Gaudreau and Blondin, 2002) is a questionnaire that contains 39 items measuring athletes’ coping strategies in competition. Consistent with previous research (Gaudreau and Blondin, 2002; Nicolas et al., 2011), the 10 subscales of the CICS were organized in the three second-order dimensions of TOC (task-oriented coping: mental imagery, thought control, effort expenditure, seeking support, logical analysis, and relaxation), DtOC (distraction-oriented coping: mental distraction and distancing), and DgOC (disengagement-oriented coping: distraction-oriented coping venting of unpleasant emotions and disengagement/resignation). Previous research has shown the validity and reliability of the CICS scores (Gaudreau and Blondin, 2002). Each item was rated on a 5-point Likert scale ranging from 1 (does not correspond at all) to 5 (corresponds very strongly). Cronbach alphas ranged from 0.73 to 0.88 (**Table 2**).

### Procedures

The study protocol has received the approval from the University of Burgundy institutional research ethics board and by the INSEP’s executive committee. The athletes’ coaches were contacted to obtain permission to approach their athletes about participation in the study.

The athletes’ participation was voluntary and written informed consent was obtained from each individual prior to data collection (as well as from the parents of underage athletes).

### Data Analyses

The data were examined for multivariate outliers and multicollinearity of scales. In order to improve our reliance in the stability of the cluster solution, hierarchical and non-hierarchical cluster analyses were carried out independently for the two waves on the standardized DSQ-60 scores using a two-step process (Vaillant, 1994). First, a hierarchical cluster analysis (Ward’s linkage with squared Euclidian distance) was carried out to define the number of clusters. Second, a *k* mean indicates that cluster analysis was conducted by mentioning the most appropriate cluster solution from stage one. We then conducted a series of MANOVAs with demographics (age, years of playing experience, and hours of training per week) and other key variables (coping, perceived stress and perceived control) entered as the dependent variables in order to explore differences between cluster groups. When relevant, *post hoc* comparisons of group means (Tukey’s HSD) using Bonferroni adjustment (*p* < 0.017 and 0.01 for α = 0.05 for demographic and external variables, respectively) were conducted; the partial eta squared (η^2^) was computed. We also carried out a series of chi-square tests of association to determine if cluster groups were confounded with sex. Finally, we performed a chi-square test of association – T1 profiles × T2 profiles – to explore whether the same participant fitted to the same coping profiles over time.

## Results

### Defense Profiles

**FIGURE 1:**
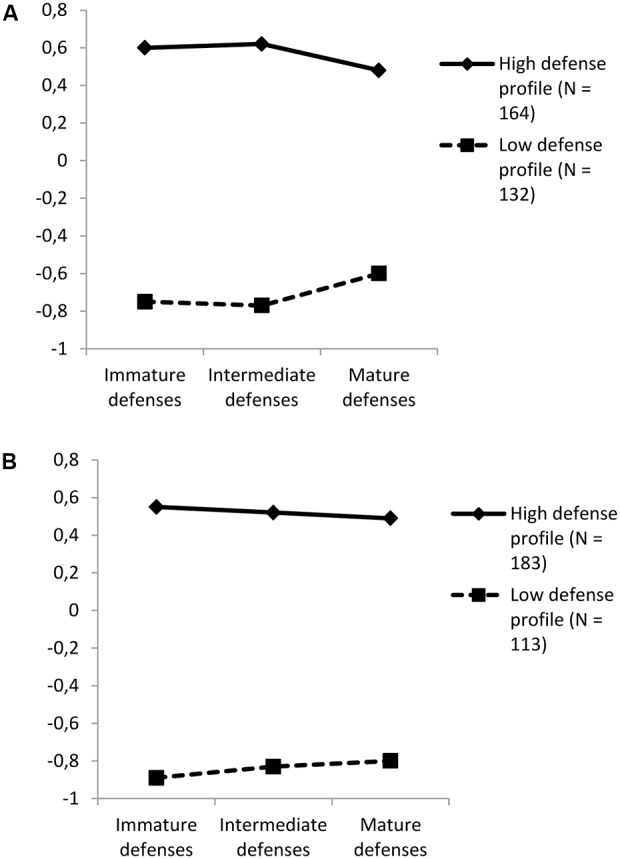
DSQ-60 standardized estimates of the defense profiles before **(A)** and during **(B)** sport competition.

Although two possible solutions were suggested by the agglomeration schedule coefficient and the dendrogram (i.e., two or three clusters), a two-cluster solution was selected for the two waves. This decision was based on empirical (e.g., number of participants in each group) and conceptual considerations (e.g., interpretability of the cluster solution). Then, a *k* mean cluster analysis (non-hierarchical cluster analysis) was performed by specifying a 2-cluster solution for the two waves. Providing the robustness of the cluster solutions for both time, a series of ANOVAs revealed that participants from the two clusters significantly differed on the three defense dimensions for the two waves (see **Table 1** for more details). **Figure 1** presents the descriptive statistics for the two clusters and for the two waves. It is noteworthy that the two clusters were almost identical across the two time points. In particular, athletes from the high defense profile (*N* = 164 and 183 for T1 and T2, respectively, 32 and 34% of female athletes, 53 and 56% of team sport athletes) reported high scores of immature, intermediate and mature defenses. In contrast, athletes from the low defense profile (*N* = 132 and 113 for T1 and T2, respectively, 35 and 32% of female athletes, 67 and 65% of team sport athletes) reported low scores of immature, intermediate and mature defenses (see **Table 1** for more details).

### Cluster Group Differences for Coping, Perceived Stress and Perceived Control

A MANOVA indicated a significant multivariate effect of clusters on the dependent variables as a whole for both measurement points. Results of *post hoc* comparisons (Tukey’s HSD) of the follow-up ANOVAs are presented in **Table 2**. For time 1, athletes from the high defense profile reported significantly higher scores of task-oriented coping, DtOC, disengagement-oriented coping, perceived stress and control than athletes from the low defense profile. For Time 2, athletes from the high defense profile reported significantly higher scores of task-oriented coping, DtOC, disengagement-oriented coping, and perceived stress than athletes from the low defense profile.

### Cluster Group Differences on Demographic Variables

A MANOVA revealed a significant multivariate effect on the demographic variables (age, years of playing experience, and hours of training per week) as a whole for Time 2, with Wilk’s Lambda = 0.97, *F*(3,288) = 3.27, *p* = 0.02, η^2^ = 0.03, but not for Time 1, with Wilk’s Lambda = 0.99, *F*(3,288) = 0.91, *p* = 0.44, η^2^ = 0.01. However, subsequent ANOVAs revealed that none of the demographic variables were significantly different across Time 2 clusters (all *ps* > 0.017). Moreover, results of chi-square tests of association showed that the number of male and female athletes was not significantly different across clusters for Time 1, χ^2^(1) = 0.33, *p* = 0.57, or Time 2, χ^2^(1) = 0.13, *p* = 0.72.

### Composition of Cluster Groups for the Two Waves

The finding of a chi-square test for Time 1 profile (2) × Time 2 profile (2) showed that the distribution of the clusters differed across Times 1 and 2 [χ^2^(1) = 85.56, *p* < 0.001] clusters. The compositions of the defense profiles over the two waves are indicated in **Table 3**. Overall, a large majority of athletes reported stable defense profiles between Times 1 and 2. More precisely, 85% from the high defense profile and 67% from the low defense profile remained in the same clusters before and after the sport competitions^1^.

**Table 3:** Change in the composition of cluster groups between the two waves.

T1 clusters	T2 clusters	Number of	Proportion of T1	Proportion of T2
		athletes	clusters	clusters
High defense profile	High defense profile (*N* = 183)	139^∗^	84.76%^∗^	75.96%^∗^
(*N* = 164)	Low defense profile (*N* = 113)	25	15.24%	22.12%
Low defense profile	High defense profile (*N* = 183)	44	33.33%	24.04%
(*N* = 132)	Low defense profile (*N* = 113)	88	66.67%	77.88%

*^∗^Over the 164 athletes composing the high defense profile (T1 cluster), 139 athletes (or 84.76% of these 164 athletes) also belonged to the high defense profile at T2. These 139 athletes represented 75.96% of the T2 high defense profile.*

## Discussion

The purpose of this study was to study the defenses of athletes in order to offer a robust heuristic for the examination of defenses in a more holistic approach. Moreover, the current study aimed to better understand the complex association between defenses and other key sports-related outcomes (coping, perceived stress and control) within a naturalistic stressful situation at two points in time (before and during competition). In addition to providing descriptions of athletes’ defense profiles, the findings of the present study indicate that (a) contrary to our expectations, two clusters of defenses were identified: high defense (i.e., participants who had reported high scores of immature, intermediate and mature defenses) and low defense (i.e., participants who had reported low scores of immature, intermediate and mature defenses); (b) the aforementioned clusters are stable before and during the sport competition; (c) athletes generally use the same defensive patterns before and after sport competitions; and (d) athletes from the high and low defense profiles differed on coping, perceived stress, and perceived control.

A limitation of previous studies was that researchers generally examined the bivariate relationships between defenses and other variables. This approach neglects the multivariate nature of defenses, as individuals may use multiple defense mechanisms in response to a particular achievement-related demanding situation such as a sport competition. Across two measurement points and clustering methods, two multivariate defense profiles were uncovered at Time 1 (before the competition) and confirmed at Time 2 (during the competition) in this sample of competitive athletes. This, in turn, provides evidence for the robustness of this two-cluster solution of high and low defense profiles. Unexpected defense profiles were uncovered for the two waves, with high defense characterizing athletes with high scores of immature, intermediate and mature defenses, and low defense profiles including low scores in immature, intermediate and mature defenses. The high defense cluster characterized individuals who appear to be oriented toward problem-solving strategies (mature defense), who manifest altruistic and prosocial behaviors (intermediate defense), and who express affect through withdrawal or acting-out (immature defense); the opposite was true for the low defense cluster. These defense dimensions, however, are not mutually exclusive. The stressful nature of sport competitions might increase the use of all defenses, including those that are mature, intermediate and immature. Future research should examine if high and low defense users could be identified in other stressful situations.

The results of this study indicate that multivariate defense profiles can emerge in achievement-related situations (e.g., sport competitions) when all defense dimensions are considered together. This offers an alternative perspective in which a combination of defense dimensions (i.e., high scores on mature and immature defenses) may serve as an additive function and would be more beneficial than only having high mature defense. These findings are consistent with other research on coping mechanisms that does not support the idea that only one coping strategy is appropriate for a specific situation. Many empirical studies have indeed provided evidence that individuals employ several coping strategies in response to a specific stressor (Matheson and Anisman, 2003; Skinner et al., 2003; Nicholls and Polman, 2007). In fact, combined use of different coping strategies is associated with a reduction in stress levels (Nicholls and Polman, 2007), better academic success (Sideridis, 2006), and consequently, appears to promote adaptation in the academic field. In addition, a cluster analysis in the sports field identified multivariate coping profiles using both TOC and DgOC (Gaudreau and Blondin, 2002) and their relationships to adaptive outcomes, by such confirming that multivariate coping profiles are related to the adaptation process.

Thus, our findings support the idea that the several defenses used by individuals during a particular achievement-related demanding situation should not be seen as independent or mutually exclusive, but rather part of a larger interconnected defense system activated by a stressful situation (Kwon and Lemon, 2000). A central tenet of defensive functioning is that individuals use different defenses based on if the stressor is internal or external (Perry et al., 2015), and that the more the person experiences stress, the more it results in an increase of defense use (Cramer, 2008). Our findings indicate that the adaptiveness of defenses is primarily based on the quantity of defenses used rather than the type of defense (adaptive or maladaptive). In other words, at a certain level, defenses aid to manage stress, however, when used excessively, defenses can cause maladaptation and even psychopathology (Cramer, 2008).

In addition to propose a description of naturally occurring combinations of defense dimensions, this study also investigated the stability/change of defense profiles over time and across demographic characteristics. Firstly, it is noteworthy that defense profiles did not differ across age, years of playing experience, and hours of training per week, demonstrating that demographic variables do not have an impact on defense configurations. Second, it is important to outline that defense profiles were comparable across the pre- and intra-competitive phases of the competition; therefore athletes generally use the same defensive patterns before and after sport competitions. Thirdly, a large majority of individuals from the high defense (i.e., 85%) and low defense (i.e., 67%) profiles have exhibited a stable coping profile across waves. Nevertheless, results indicated that the distribution of athletes in the two defense profiles significantly varied across phases of the competition. More precisely, several athletes in the precompetitive high defense (i.e., 15%) and low defense (i.e., 33%) profiles did not belong to the same defense profiles before and during the competition. At Time 2, there were twice as much change in low defense profile and more athletes in the high defense profile after the competitions. In this specific situation of sport competition, challenges and demands inherent to sport competition (Cerin et al., 2000) might explain the small variations before and after the event. Overall, these findings indicate the usefulness of longitudinal designs that enable researchers to apprehend variations in specific combinations of defenses across phases of a sport competition. Despite these changes, the defense clusters remained stable across the phases of the sport competition. This is congruent with the literature that conceptualizes defenses as a trait (Kernberg, 1984; Endler and Parker, 2002; Bouchard and Thériault, 2003; Nicolas and Jebrane, 2008, 2009; Cramer, 2015).

The two clusters of high and low defense differed significantly for all of the coping strategies (TOC, DtOC, DgOC) and perceived stress at the two time points (before and after the competitions), and for perceived control at T1 before the competitions (see **Table 2**). Compared to athletes from the low defense profile, athletes from the high defense profile used a significantly greater number of all the dimensions of coping strategies and experienced higher levels of perceived stress and control. The context may have an important impact on these defense patterns, despite the fact that defenses are considered relatively stable and closely related to the individual’s personality (Kernberg, 1984). Hence, contexts that are achievement driven and highly competitive might influence an individual’s defense configurations to a certain degree. These findings underline the benefit of an ideographic approach (i.e., person-centered assessment of defense profiles) compared to a nomothetic approach (i.e., standard variable-centered approach), as an ideographic approach suggests specific combinations of defenses and their idiosyncratic consequences for the key sport outcomes. However, although the scores of the high defense cluster were significantly higher than in the low defense in mature, intermediate and immature dimensions, the scores remained at a certain level and under the arithmetic mean of the Likert-scale, which suggests that the threshold was relatively low and not clinically relevant.

Taken together, the current study highlights the role of defenses in a non-pathological population during particular achievement-related situations, such as a sport competition. These findings are consistent with both theory and the few previous empirical studies in this area (Fulde et al., 1995; Erickson et al., 1997; Bouchard and Thériault, 2003; Nicolas and Jebrane, 2008). Given the contribution of defenses to the adaptation process, interventions should not only be based on coping but on defenses as well (Nicolas and Jebrane, 2009; Drapeau et al., 2011). In order to adapt interventions to the individual athlete’s abilities and to improve their adaptation process in sports, psychologists and sport consultants should develop the athletes coping repertoire according to their dispositions and more particularly to their individual defense profiles (Nicolas and Jebrane, 2009). Thus, techniques of mental preparation (e.g., cognitive reappraisals, emotion regulation, goal setting) should be adapted to the characteristics of the athletes and not the contrary.

### Limits

One criticism of the assessment of defense mechanisms using self-report measures (quantitative methods) is that defenses are largely unconscious processes, thus are difficult to measure with questionnaires. Defenses were evaluated in the present study with the DSQ which is intended to assess conscious derivatives of defenses (Bond, 2004). Although the DSQ has been revised, validated and translated in numerous languages, the utilization of self-report to evaluate defense remains controversial. Clinical measures provide a more direct assessment of defensive activity (Andrews et al., 1989), however, they entail greater effort to (a) reduce subjectivity, (b) gain adequate reliability, and (c) investigate a representative number of participants. Thus, the use of clinical assessment by external observers to assess defenses (Cramer, 2015) in future studies would be an important extension to the current findings. A second limitation in this study lies in the type of data analysis performed. Given the main goal of this study was to examine the concept of defense profiles (i.e., naturally occurring configurations of defenses) and its relationship with theoretically relevant variables (i.e., coping, perceived stress and control), we used a cluster analysis approach. Therefore, despite the temporal design, the potential temporal effects between the study variables were not explored especially with the limits of measuring psychological states within 2 h before and after the event. Future research should further explore the temporal relationship between defenses, coping, perceived stress and control within sport competition (Nicolas et al., 2017).

## Conclusion

Despite some limitations, the findings of the present study contribute to our knowledge of the psychological adaptation process that occurs during sport competition. The findings of the current study indicate that there may be stable defense profiles both before and during sport competition and differences in coping, stress and control. Athletes with high defense profiles reported higher levels of coping strategies, perceived stress and control than athletes with low defense profiles, confirming that defenses are involved in the psychological adaptation process. These findings have theoretical consequences for researchers and practical implications for sport psychologists and practitioners, as they adapt their mental preparation to the athletes’ individual defense profile in order for example to increase mature instead of immature defenses. Given the contribution of defenses to the adaptation process, it is apparent that research and intervention should not be based solely on coping, but rather on defenses as well (Nicolas and Jebrane, 2008; Drapeau et al., 2011).

## Author Contributions

MN carried out the data collecting and supplied the original version. GM realized the statistical data processing. Finally, GM, MD, KC, PV, and YdR participated to the manuscript redaction.

## Conflict of Interest Statement

The authors declare that the research was conducted in the absence of any commercial or financial relationships that could be construed as a potential conflict of interest.

## Footnotes

1 In order to further explore whether athletes who switched cluster membership had significantly different values for defense style at T1 (i.e., immature, intermediate, and mature defenses), we conducted two MANOVAs (i.e., one for athletes of the high defense profile at T1 and the other for athletes of the low defense profile at T1) with T1 defense scores as dependent variables. Results of MANOVA conducted for athletes belonging to the T1 high defense profile were non-significant, indicating that athletes who stayed in this cluster at T2 did not significantly differed on T1 defense scores as a whole from athletes who switched in the low defense profile at T2, Wilks’s Λ = 0.96, *F*(3,160) = 2.01, *p* > 0.10, η^2^ = 0.04. In contrast, results of MANOVA conducted for athletes belonging to the T1 low defense profile were significant, indicating that athletes who stayed in this cluster at T2 significantly differed on T1 defense scores as a whole from athletes who switched in the high defense profile at T2, Wilks’s Λ = 0.93, *F*(3,128) = 3.14, *p* = 0.03, η^2^ = 0.07. The results of univariate ANOVAs indicated that only the scores of immature defenses significantly differed across athletes who stayed in the low defense profile at T2 (*M* = -0.87; *SD* = 0.66) and those who switched to the high defense profile at T2 (*M* = -0.51; *SD* = 0.74), *F*(1,130) = 7.79, *p* = 0.006, η^2^ = 0.06.
